# New insights from the widening homogeneity perspective to target intratumor heterogeneity

**DOI:** 10.1186/s40880-018-0287-y

**Published:** 2018-05-04

**Authors:** Mengying Tong, Ziqian Deng, Xiaolong Zhang, Bin He, Mengying Yang, Wei Cheng, Quentin Liu

**Affiliations:** 1 Institute of Cancer Stem Cell, Cancer Center, Dalian Medical University, Dalian, 116044 Liaoning P.R. China; 2 State Key Laboratory of Oncology in South China, Sun Yat-sen University Cancer Center, Guangzhou, 510630 Guangdong P.R. China; 3 Department of Hematology, The Third Affiliated Hospital, Sun Yat-sen Univerisity, Guangzhou, 510630 Guangdong P.R. China

**Keywords:** Acquired drug resistance, Homogenization, Intratumor heterogeneity, Plasticity, Precision medicine

## Abstract

Precision medicine has shed new light on the treatment of heterogeneous cancer patients. However, intratumor heterogeneity strongly constrains the clinical benefit of precision medicine. Thus, rethinking therapeutic strategies from a different facet within the precision medicine framework will not only diversify clinical interventions, but also provide an avenue for precision medicine. Here, we explore the current approaches for targeting intratumor heterogeneity and their limitations. Furthermore, we propose a theoretical strategy with a “homogenization” feature based on iatrogenic evolutionary selection to target intratumor heterogeneity.

## Background

Tumor heterogeneity includes intertumor and intratumor heterogeneities. Genetic and phenotypic variations are observed among different tumor patients [1, 2]. Extremely high genetic diversity makes each patient unique and distinct. However, within a tumor, both genomic instability and the tumor subclone architecture vary over time [3–8]. As the tumor evolves, the parental subclone acquires an increasing number of genetic and epigenetic alterations, resulting in a tumor with different subclone phenotypes.

Intratumor heterogeneity is characterized by its dynamic changes. Tumor initiation and progression are generated from stochastic to sequential mutations that contribute to subsequent clonal expansion and intratumor heterogeneity [9]. Therefore, a single biopsy is unlikely to capture the complete genomic landscape of a patient’s tumor, considering the spatiotemporal changes in tumor heterogeneity [10]. Consequently, even if the subclone harboring the detected molecular phenotype has been targeted effectively, other subclones of the tumor may still grow. Moreover, the sensitive subclone may become resistant to therapy, causing further disease deterioration.

Tumor homogeneity refers to the cellular populations bearing the same or similar genetic or epigenetic characters within the same lesion or in different lesions of the same patient. Here, we propose an ideal situation in which the tumor becomes a homogeneous cell population, i.e., tumor cells that acquire common molecular properties. Once tumor heterogeneity is drastically confined in this manner, the cells are susceptible to a single intervention that targets this particular feature. Recent technological advances in both molecular diagnostics and targeted drugs have led to the theory of “acquiring tumor homogeneity”.

This review summarizes the recent understanding and clinical practice of precision therapy, and illustrates the current strategies and limitations for targeting intratumor heterogeneity. Then, we discuss the possibility and implementation of “homogenization” therapy for precision medicine.

## Intratumor heterogeneity challenges precision medicine

Precision therapy exerts profound effects on cancer patients. Sequencing technology and genomic analyses are driving the progress of precision therapy. In the clinic, molecular diagnosis has been applied to biopsies of tumor tissues to guide the selection of precision therapy [11, 12]. However, the outcomes of clinical trials regarding the assessment of precision therapy are discouraging. For example, the SHIVA trial showed no significant difference in progression-free survival (PFS) between targeted and conventional therapies [13]. The Princess Margaret IMPACT-COMPACT study reported a non-randomized comparison, which indicated an objective tumor response rate of 20% in the matched group (between genotype and targeted therapy) versus 11% in the unmatched group [14].

### Intratumor heterogeneity and plasticity

Precision therapy has been hindered by multiple factors, resulting in the limited success of clinical therapy. Tannock et al. reviewed the problem and concluded that Darwinian evolution leading to intratumor heterogeneity may weaken the effect of precision therapy [15]. Genetic alterations, including aneuploid rearrangements and point mutations, generate extensive clonal diversity [16]. A recent study [17] showed that more than 100 million coding region mutations exist in a single tumor, and such high genetic variation subverts the effect of targeted therapy. Moreover, genetically [18, 19] and epigenetically unstable tumorigenic cells contribute to tumor plasticity. Evidence from both cell line [20–23] and animal model [24, 25] indicates that tumorigenic cells display cellular plasticity that allows them to transit between different states. Overall, intratumor heterogeneity and plasticity co-exist within a tumor, and the unification of which comprehensively illustrates the difficulty of precision therapy.

**Fig. 1:**
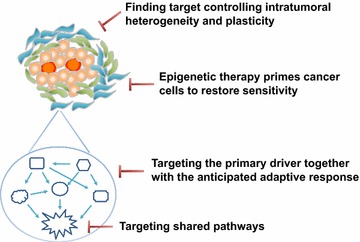
Current strategies targeting intratumor heterogeneity. Four strategies to solve this problem are as follows. First, targeting a shared pathway. Second, targeting the primary mutation together with the anticipated mutation. Third, finding a target controlling intratumor heterogeneity and plasticity. Fourth, epigenetic therapy that primes cancer to restore sensitivity

Based on the current knowledge of intratumor heterogeneity and plasticity, two strategies have been proposed to partly solve the problem (Fig. 1). First, targeting a shared pathway may be practical when parallel mutations leading to pathway convergence are detected [26]. For example, different molecular subtypes of breast cancer share pathways including Notch [27], Wnt [28], Her-2 [29], and STAT3-NF-kB [30]. In renal cancer, constraints in activation of the PI3K/mTOR pathway, which manifests as the shared pathway of mutations in PTEN, PIK3CA, TSC1, or mTOR, might be exploitable for therapeutic benefit [31]. Therefore, despite the diversity of numerous mutations, these mutations affect the same pathways, and agents that target these pathways may maximize the benefit of precision therapy [32–34].

The second strategy is to block cellular plasticity by preventing the transition between cellular states, which may increase therapeutic efficacy. This strategy includes inhibitors of c-Met [35] and TGF-β [36]. Moreover, two recent studies [24, 25] showed that mutant PIK3CA in breast cancer induces multipotency in lineage-committed basal and luminal cells, which drives plasticity and intratumor heterogeneity. Likewise, in pancreatic cancer, PI3K/PDK1 signaling pathway mediates cellular plasticity and acts as a key effector of oncogenic Kras [37]. An interesting recent study [38] suggested that p53 is essential for DNA methylation homeostasis in embryonic stem cells, and the loss of which promotes clonal heterogeneity. Taken together, identifying the mechanisms contributing to intratumor heterogeneity is imperative to identify suitable targets for therapeutic interventions.

### Intratumor heterogeneity and acquired drug resistance

Therapeutic intervention can induce a drug-tolerant phenotype in the absence of a pre-existing resistant clone [39, 40]. A recent study demonstrated that application of a drug initiates cellular reprogramming, revealing a mechanism of acquired drug resistance [41]. Consequently, continued targeted therapy in the presence of resistant subclones might accelerate tumor progression [42]. For example, continued BRAF inhibitor treatment results in tumor metastasis of RAS- and BRAF-mutant melanoma cells [43] and paradoxical activation of the RAS-ERK pathway in multiple myeloma clones [44]. Therefore, blind or persistent use of targeted therapy for a drug-resistant tumor is inappropriate. Accurate and timely monitoring of the evolving molecular landscape of a tumor is critical but difficult.

Currently, substantial efforts have attempted to solve the problem of intratumor heterogeneity and acquired drug resistance (Fig. 1). The first approach is to target the primary driver and simultaneously block the anticipated adaptive response [32, 45]. For example, in breast cancer, MAPK can be activated in response to PI3K inhibition. Thus, the combination of MEK and PI3K inhibitors shows great potential [46–50]. Likewise, in BRAF-mutant melanoma, resistance to BRAF inhibitors is mediated by reactivation of MAPK [43, 51] and PI3K-PTEN-AKT [51] pathways. Thus, preemptive inhibition of the MEK pathway [52, 53] or both MAPK and PI3K pathways [51] can prolong PFS. However, because epigenetics play an important role in cellular plasticity and drug resistance [54–57], epigenetic therapy has gradually gained popularity to sensitize tumor cells for therapy. For example, DNA methylation and histone deacetylase inhibitors are thought to prime cancer cells to restore sensitivity to previously ineffective drugs [58, 59].

## Exploring the “homogenization” strategy for precision medicine

**Fig. 2:**
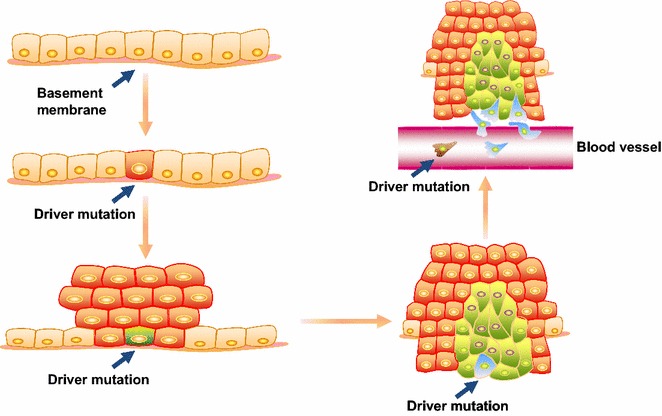
Driver mutations in tumor evolution. A cell develops a specific driver mutation that gives rise to a benign tumor. The cell then develops an additional driver mutation to invade surrounding tissues. Subsequently, the cell develops an additional driver mutation that enables the tumor to engage in hematogenous metastasis. Thereafter, the tumor may acquire additional driver mutations to propagate and sustain intratumor heterogeneity

**Fig. 3:**
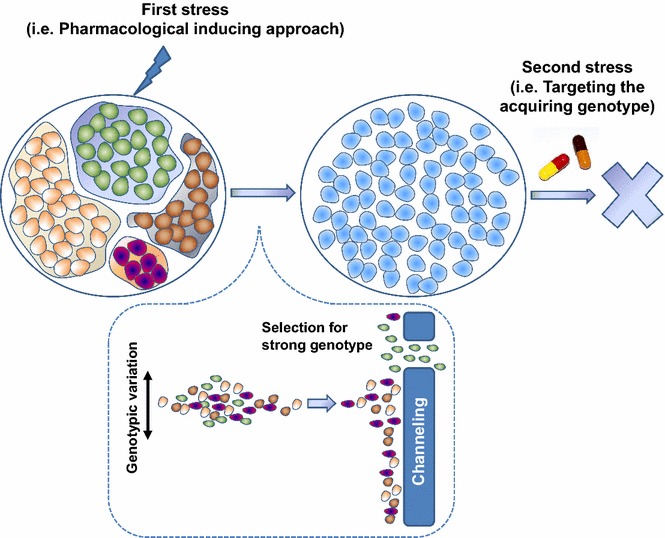
Schematic depiction of “homogenization” therapy. First, a homogeneous cell population is induced, followed by administration of a drug to which the cells are sensitive to eradicate the tumor population

Limitations exist in all approaches mentioned above regarding targeting intratumor heterogeneity. Considering the complex signaling pathways, identifying the driver gene contributing to intratumor heterogeneity is difficult. Evolution from a tumor cell to an advanced cancer is a long process, and a large number of driver genes are involved in further deterioration (Fig. 2). Therefore, we propose an alternative approach to target intratumor heterogeneity. The “homogenization” strategy introduces a consistent genotype or creates a specific environment to drive all tumor cells exhibit the same phenotype. This approach weakens intratumor heterogeneity and results in a less diverse cell population. Thereafter, administration of a sensitive drug that targets these cells would eliminate the tumor population (Fig. 3).

### Principles of the homogenization strategy

Because the tumor cell population is highly heterogeneous and unstable, evolutionary dynamics can be capitalized to select a homogenous cell population under stress. As the tumor evolves, unadapted subclones are completely lost, whereas fit subclones become dominant, and less fit minor subclones persist by forming reservoirs from which evolution can continue [60]. Consequently, therapeutic interventions may eliminate specific clones and inadvertently exert selective pressure on the propagation of resistant clones [39, 40]. Thus, homogenization imposes selective pressure on tumor cells with genetic diversity by eliminating the therapy-sensitive cell population, leading to a therapy-resistant cell population with a highly adaptive potential. Genetically, this process strongly accelerates and exacerbates a particular mutation deficiency, which emerges as an adaptive variant. Thus, surviving cells have a common and predominant genotypic variation.

To drive tumor cells into a homologous population, a selective condition must to be identified for homogenization. A general pharmacological selection approach to gain a cell population with shared adaptability and fitness from a heterogeneous cell population is outlined here. In this case, exploiting iatrogenic evolutionary selection pressure is tractable and collateral sensitivity emerges in general, i.e., sensitivity to another drug at the expense of resistance acquired to one drug has been observed [61, 62]. Such evolutionary constraints have been exploited in a murine model of acute lymphoid leukemia [63]. Specifically, dasatinib treatment results in selection of the acquired resistance BCR-ABL1 V299L mutation and renders cells sensitive to non-classical BCR-ABL inhibitors such as cabozantinib and vandetanib.

### Techniques and resources facilitate implementation of a second stress

After selection of a homogenous tumor cell population via adaptation, identification of the selected genotype is required. Based on our understanding, three possibilities can be considered. First, a target gene likely exists, and genetic variation of the original drug target of the targeted gene is possible. Second, a particular genotype is functionally associated with activation of the downstream signaling pathway of the original target gene, which provides a possibility to bypass the first stress. Third, the emerging genotype of the certain gene probably locates near the original target gene in the genome. Therefore, the genotype induced by the first stress can be determined by targeted deep sequencing of these three possibilities.

With the use of targeted deep sequencing, oncologists can profile the spectrum of genomic changes in a tumor sample. For example, a typical study revealed the molecular taxonomy of prostate cancer by omics analysis as well as potentially actionable targets [64]. Moreover, research initiatives, such as The Cancer Genome Atlas (TCGA) and International Cancer Genome Consortium (ICGC), capitalize the available information of large numbers of tumor samples to identify genes and pathways important for cancer progression [65–69]. Targeted drugs, which are designed to specifically suppress certain oncogenic signaling pathways, are widely applied in clinical practice. Thus, precision therapy is appealing and changing the pattern of clinical practice for tumor patients. Notably, a multicenter and prospective study called tracking cancer evolution through therapy (TRACERx) [70–73] provided an applicable model to track tumor evolution depending on multiregion and longitudinal sampling and genetic analysis. Going forward, such large scale genomic studies may contribute to clinical practices.

Subsequently, there is a need to determine a drug that specifically targets the newly acquired genotype through drug screening or designing a targeted drug. To this end, cancer systems biology may provide a more holistic view of cancer [74, 75]. Specifically, this approach can bridge molecular characteristics with pharmacogenomics to deliver targeted therapy, which will significantly improve the specificity and efficacy of targeted therapy. Moreover, some datasets [76] are rich resources to identify therapeutic options for selected targets.

### Dynamic monitoring of the homogenization genotype

The implementation of dual drug stresses theoretically leads to the extinction of the tumor cell population. Nevertheless, the theory of evolutionary dynamics proposes that the homogenous cell population can gain the ability to escape the second stress via continued phenotypic changes. To solve this problem, the genotype induced by the first stress should be monitored to avoid the emergence of resistance to the second drug. When a reduction is detected, the first drug should be used again to select and enrich the particular genotype, which makes the selected cell population more vulnerable to the second drug. Furthermore, when an increase is detected, therapy with the second drug should be resumed. The opposing selective effect of these two drugs imposes an adaptive dilemma for the selected homogenous genotype, and an analogous strategy has been demonstrated by many studies [77–81].

Current techniques that facilitate dynamic monitoring of the homogenization genotype include liquid biopsies [82–85] such as circulating tumor cells (CTCs) and circulating free DNA (cfDNA), which allows assessment of repeated samples and longitudinal measurement of clonal evolution in tumor patients. Conceivably, combining omics sequencing with liquid biopsies is required for dynamic monitoring.

## Future perspectives

Homogenization therapy is based on inducing a common and druggable characteristic trait of a heterogeneous cell population. The genetic representation of clonal evolution from the primary tumor to relapse reveals convergent evolution, suggesting that this strategy is possible [86]. In addition, a proof-of-principle study in yeast has demonstrated the feasibility of this strategy [87]. Specifically, an “evolutionary trap” has been devised by reducing karyotypic heterogeneity to a defined predictable state via initial drug exposure, and then a secondary drug was subsequently applied. This study illustrated that evolutionary dynamics can be exploited for homogenization therapy, and evidence is emerging to support the efficacy of this approach. A case study [81] has reported that a patient with ALK-rearranged non-small-cell lung carcinoma, which harbored a subclonal C1156Y mutation, acquired drug resistance to crizotinib and responded to the third-generation ALK inhibitor lorlatinib. Following treatment with lorlatinib, the tumor acquired an L1198F mutation, but this mutation promoted resensitization to crizotinib, which improved the patient’s prognosis. Ultimately, other options and interventions to achieve homogeneity should be explored further. Moreover, exploration of tumor homogeneity requires the design of more clinical practices. Taken together, homogenization therapy sheds new light on our understanding of intratumor heterogeneity and provides a novel strategy to solve problems associated with tumor treatments.
